# Draft Genome Sequences of Interpatient and Intrapatient Epidemiologically Linked Neisseria gonorrhoeae Isolates

**DOI:** 10.1128/genomeA.00319-18

**Published:** 2018-04-19

**Authors:** Sonja Hirk, Sarah Lepuschitz, Adriana Cabal Rosel, Steliana Huhulescu, Marion Blaschitz, Anna Stöger, Silke Stadlbauer, Petra Hasenberger, Alexander Indra, Daniela Schmid, Werner Ruppitsch, Franz Allerberger

**Affiliations:** a Institute of Medical Microbiology and Hygiene, Austrian Agency for Health and Food Safety, Vienna, Austria; b European Public Health Microbiology Training Programme (EUPHEM), European Centre for Disease Prevention and Control (ECDC), Stockholm, Sweden

## Abstract

Neisseria gonorrhoeae is the causative agent of gonorrhea and was identified by the World Health Organization as an urgent public health threat due to emerging antibiotic resistance. Here, we report 13 draft genome sequences of N. gonorrhoeae isolates derived from two epidemiologically linked cases from Austria.

## GENOME ANNOUNCEMENT

Neisseria gonorrhoeae is the etiological agent of the sexually transmitted disease gonorrhea, and it poses a public health threat due to the emergence of multidrug-resistant strains (1–4). Whole-genome sequencing is considered a powerful strategy to elucidate chains of transmission (5). Here, we announce the draft genome sequences of 13 epidemiologically linked N. gonorrhoeae isolates.

Two vaginal swabs, taken from a 3-year-old girl on 10 January 2018, and her rectal swab, gained on 13 January 2018, yielded N. gonorrhoeae colonies on Chocolat PolyViteX VCAT3 agar plates (bioMérieux, Marcy-l’Étoile, France). A 46-year-old male household member was sampled on 13 January 2018, and N. gonorrhoeae colonies grew from a rectal swab. Eight single colonies from the child and five from the adult were further analyzed.

For each isolate, antimicrobial susceptibility was determined according to the EUCAST recommendations for gonococci (6). All 13 isolates showed resistance to penicillin G (median MIC, 6 µg/ml; range, 1.5 to 32 µg/ml), tetracycline (median MIC, 24 µg/ml; range, 24 to 64 µg/ml), and ciprofloxacin (median MIC, 0.75 µg/ml; range, 0.5 to 1.5 µg/ml), but were susceptible to ceftriaxone, cefixime, and azithromycin.

Genomic DNA isolation, whole-genome sequencing, assembly, and contig filtering were performed as described previously (7). Paired-end sequencing (2 × 300 bp) generated 348,172 to 847,328 reads, with a mean coverage of 41- to 89-fold. The NCBI Prokaryotic Genome Automatic Annotation Pipeline identified 2,654 to 2,720 genes, 2,604 to 2,664 coding sequences, 273 to 305 pseudogenes, 3 to 6 rRNA genes, and 47 to 51 tRNA genes.

Antimicrobial resistance genes were identified using the Comprehensive Antibiotic Resistance Database (CARD) (8). All 13 isolates had *gyrA*, N. meningitidis PBP2 and *rpsJ,* and the efflux genes *farA*, *farB*, *macA*, *macB*, *mtrC*, *mtrD*, and *mtrR*. In addition, *bla*_TEM-1_ was detected in three child and three household member isolates. Three isolates from the child and one from the household member carried *bla*_TEM-90_. One child isolate had *bla*_TEM-150_, and another one carried *bla*_TEM-150_ plus the efflux gene *patA*.

All 13 isolates belonged to multilocus sequence type (MLST) 1588 (ST1588). An *ad hoc* core genome MLST (cgMLST) scheme comprising 1,524 targets was established using strain MS11 (ATCC BAA-1833) as a reference. Child isolates differed by zero to three alleles and household member isolates by zero to one alleles; the maximum interindividual variability of the isolates was five allelic differences. In the course of comparison with the Austrian Agency for Health and Food Safety (AGES) N. gonorrhoeae whole-genome database (currently covering 452 isolates from the years 2014 to 2018), all but one isolate differed by at least 303 alleles. An isolate gained in 2016 (strain 980016-16) from a urethral swab of an epidemiologically unrelated 32-year-old male patient, registered in the same Austrian province as the two described case patients, showed a six-allele difference. From these results, we propose a complex-type threshold of a maximum of five allelic differences for direct transmission events of N. gonorrhoeae. Our findings underline the considerable potential of whole-genome sequencing (WGS) to document chains of transmission.

### Accession number(s).

This whole-genome shotgun project has been deposited at DDBJ/EMBL/GenBank under the accession numbers shown in Table 1. The versions described in this paper are the first versions.

**TABLE 1:** Fourteen N. gonorrhoeae isolates included in BioProject PRJNA433931

Strain	GenBank accession no.	No. of contigs	Total length (bp)
980035-18	PTPT00000000	173	2,234,140
980036-18	PTPS00000000	155	2,227,585
980037-18	PTPR00000000	203	2,214,439
980038-18	PTPQ00000000	193	2,239,226
980039-18	PTPP00000000	164	2,214,439
980040-18	PTPO00000000	191	2,232,201
980041-18	PTPN00000000	156	2,226,960
980042-18	PTPM00000000	168	2,223,307
980043-18	PTPL00000000	160	2,217,312
980044-18	PTPK00000000	137	2,214,035
980045-18	PTPJ00000000	141	2,210,712
980046-18	PTPI00000000	140	2,217,914
980047-18	PTPH00000000	199	2,209,742
980016-16	PTPG00000000	178	2,233,553
